# Steroid Resistance in COPD? Overlap and Differential Anti-Inflammatory Effects in Smokers and Ex-Smokers

**DOI:** 10.1371/journal.pone.0087443

**Published:** 2014-02-05

**Authors:** Susan J. M. Hoonhorst, Nick H. T. ten Hacken, Judith M. Vonk, Wim Timens, Pieter S. Hiemstra, Thérèse S. Lapperre, Peter J. Sterk, Dirkje S. Postma

**Affiliations:** 1 University of Groningen, University Medical Center Groningen, Department of Pulmonary Diseases, Groningen, The Netherlands; 2 University of Groningen, University Medical Center Groningen, GRIAC research institute, Groningen, The Netherlands; 3 University of Groningen, University Medical Center Groningen, Department of Epidemiology, Groningen, The Netherlands; 4 University of Groningen, University Medical Center Groningen, Department of Pathology and Medical Biology, Groningen, The Netherlands; 5 Leiden University Medical Center, Department of Pulmonology, Leiden, The Netherlands; 6 University of Amsterdam, Academic Medical Centre Amsterdam, Department of Pulmonary Diseases, Amsterdam, The Netherlands; University of British Columbia, Canada

## Abstract

**Background:**

Inhaled corticosteroids (ICS) reduce exacerbation rates and improve health status but can increase the risk of pneumonia in COPD. The GLUCOLD study, investigating patients with mild-to-moderate COPD, has shown that long-term (2.5-year) ICS therapy induces anti-inflammatory effects. The literature suggests that cigarette smoking causes ICS insensitivity. The aim of this study is to compare anti-inflammatory effects of ICS in persistent smokers and persistent ex-smokers in a post-hoc analysis of the GLUCOLD study.

**Methods:**

Persistent smokers (n = 41) and persistent ex-smokers (n = 31) from the GLUCOLD cohort were investigated. Effects of ICS treatment compared with placebo were estimated by analysing changes in lung function, hyperresponsiveness, and inflammatory cells in sputum and bronchial biopsies during short-term (0–6 months) and long-term (6–30 months) treatment using multiple regression analyses.

**Results:**

Bronchial mast cells were reduced by short-term and long-term ICS treatment in both smokers and ex-smokers. In contrast, CD3^+^, CD4^+^, and CD8^+^ cells were reduced by short-term ICS treatment in smokers only. In addition, sputum neutrophils and lymphocytes, and bronchial CD8^+^ cells were reduced after long-term treatment in ex-smokers only. No significant interactions existed between smoking and ICS treatment.

**Conclusion:**

Even in the presence of smoking, long-term ICS treatment may lead to anti-inflammatory effects in the lung. Some anti-inflammatory ICS effects are comparable in smokers and ex-smokers with COPD, other effects are cell-specific. The clinical relevance of these findings, however, are uncertain.

## Introduction

Chronic obstructive pulmonary disease (COPD) is characterized by chronic, partially reversible airflow limitation [1]. This airflow limitation is generally progressive and associated with an inflammatory process in the airways. The main cause for the development of COPD is cigarette smoking. Smoking cessation is the most effective way to reduce disease progression and to prevent mortality [1]. Interestingly, inflammation is reduced in asymptomatic smokers who stopped smoking [2]. In contrast, there is evidence that airway inflammation persists or even increases in COPD patients after smoking cessation [2], [3].

Inhaled corticosteroids (ICS) have anti-inflammatory effects in asthma. Clinical effects of ICS in COPD have been investigated widely, providing positive effects on exacerbation rates and health status especially in advanced stages [4]–[6], but also of harm (e.g. pneumonia) [7]. The GLUCOLD study has shown that besides positive effects on lung function decline, hyperresponsiveness and health status, long-term (2.5-year) therapy with ICS additionally induces anti-inflammatory effects in COPD patients with mild-to-moderate severe disease [8]. Bronchial T-lymphocyte and mast cell numbers were reduced after treatment as well as sputum cell counts. Importantly, these anti-inflammatory effects were accompanied by a reduction in FEV_1_ decline and improvement in airway hyperresponsiveness and health status [8]. Other studies either found no anti-inflammatory effects as assessed in sputum [9], or confirmed anti-inflammatory effects of three-month ICS treatment assessed in bronchial biopsies of COPD patients, including reductions in CD8^+^ lymphocytes, mast cells and macrophages [10]–[12].

There are several indications that cigarette smoking may induce resistance to the anti-inflammatory effects of ICS. Smoking asthmatics are less sensitive to corticosteroids than non-smoking asthmatics [13]–[15]. One short-term study showed no anti-inflammatory effects by ICS treatment in smokers with COPD, whereas limited beneficial effects were observed in ex-smokers [16]. *In vitro* studies have shown that oxidative and nitrative stress, as can be caused by cigarette smoking, inactivates histone deacetylase-2, which is involved in the suppression of activation of inflammatory genes [17], [18].

The GLUCOLD study provided evidence for both clinical and anti-inflammatory effects of long-term ICS treatment in COPD. So far, no studies have linked long-term clinical and inflammatory effects of ICS treatment with active smoking in COPD. The aim of the current study is to assess anti-inflammatory effects of ICS in persistent smokers and persistent ex-smokers with COPD by a post-hoc analysis of the GLUCOLD study.

## Methods

### Ethics Statement

The study was approved by the medical etics committee of Leiden University Medical Center (LUMC) and by the medical etics committee of University Medical Center Groningen (UMCG). All patients signed written informed consent. The study is registered at ClinicalTrials.gov with identifier NCT00158847.

### Subjects

All persistent smokers and persistent ex-smokers from the GLUCOLD cohort were included [8]. To obtain the largest contrast possible, patients who switched in smoking status were excluded. In- and exclusion criteria have been described in detail previously [8]. Briefly, patients were aged 45–75 years, had a smoking history of more than ten packyears, and irreversible airflow limitation compatible with Global Initiative for Chronic Obstructive Lung Disease (GOLD) stages II and III [1]. Patients with asthma or other active lung disease were excluded.

### Study Design

Patients were randomly assigned into four groups as described before [8]: 1) fluticasone propionate (FP) 500 µg twice daily (b.i.d.) for the first six months, followed by placebo b.i.d. for 24 months; 2) FP 500 µg b.i.d. for 30 months; 3) FP 500 µg b.i.d., and salmeterol (S) 50 µg b.i.d. in a single inhaler for 30 months; 4) placebo b.i.d. for 30 months.

### Measurements

At baseline and after 6- and 30-month treatment, inflammatory cells in induced sputum and bronchial biopsies, postbronchodilator forced expiratory volume in one second (FEV_1_), and the provocative concentration of metacholine causing a 20% fall in FEV_1_ (PC_20_) were measured. Sputum samples were obtained by the full sample method [19]. Differential cell counts were expressed as a percentage of nucleated cells excluding squamous cells as previously described [19]. Fiberoptic bronchoscopy, biopsy processing and quantification were performed as described before [3]. Immunohistochemistry was performed by specific antibodies against T-lymphocytes (CD3, CD4, CD8), and mast cell tryptase (AA1) [3]. The number of subepithelial positively staining inflammatory cells was counted within the largest possible area of maximal 125 mm deep beneath the basement membrane, per biopsy section, and expressed as the mean number of cells/0.1 mm2 of the two biopsies [3]. FEV_1_ was assessed by standardized procedures as well as the two-minute tidal breathing method to measure PC_20_ methacholine [19].

### Statistical Analyses

The absolute changes between the measurements at baseline and 6 months, and between the measurements at 6 months and 30 months, were calculated for FEV_1_, PC_20_, and inflammatory cells in sputum and bronchial biopsies. These absolute changes were normalized by Ln-transformation when necessary. Multiple regression analyses were performed on these (Ln-transformed) absolute changes after short-term ICS treatment (baseline to 6 months) and after long-term ICS treatment (6 to 30 months). For the analyses from baseline to 6 months, the FP 6-month, FP 30-month, and FP/S 30-month groups were combined. For the analyses from 6 to 30 months, the placebo group was combined with the FP 6-month group, and the FP 30-month group was combined with the FP/S 30-month group. The effects of ICS treatment in persistent smokers and persistent ex-smokers and the interaction of ICS with smoking were investigated in the total population by multiple regression models. All models were adjusted for gender and age. The analyses were performed with SPSS version 18.0.3 and a p-value <0.05 was considered statistically significant.

## Results

Of the 101 patients who adhered to therapy (>70% medication use) [8], 72 patients who were persistent smokers (n = 41) and persistent ex-smokers (n = 31) were included in the current analyses. Characteristics of these 72 COPD patients among the different treatment groups are shown in Table 1 including the measurements at baseline, and after 6 or 30 months of ICS treatment.

**Table 1:** Patient characteristics.*

Treatment time (months)	0–6 months											6–30 months									
	Smokers placebo			Smokers ICS		Ex-smokers placebo			Ex-smokers ICS			Smokers placebo		Smokers ICS			Ex-smokers placebo		Ex-smokers ICS		
	0		6	0	6	0		6	0		6	6	30	6	30		6	30	6	30	
**Patients, n**	11			30		6			25			20		21			17		14		
**Men/women**	10/1			23/7		6/0			25/0			16/4		17/4			17/0		14/0		
**Age, years**	63[51–66]			59[55–64]		62[57–70]			67[62–71]			62[51–66]		58[56–64]			67[61–70.0]		69[60–71]		
**FEV**_1_, post, L	1.8[1.7–2.6]	2.0[1.5–2.5]		2.1[1.7–2.3]	2.0[1.6–2.2]	1.9[1.4–2.2]	1.7[1.5–2.1]		2.0[1.7–2.4]	2.1[1.8–2.3]		2.0[1.5–2.4]	1.7[1.3–2.2]	2.0[1.5–2.3]		1.91[5–2.4]	2.0[1.6–2.4]	2.1[1.5–2.4]	2.1[1.9–2.3]		2.1[1.9–2.3]
**PC_20_, mg/mL**	1.0[0.3–1.5]	0.3[0.3–2.2]		0.7[0.2–2.8]	1.2[0.2–7.6]	0.45[0.1–2.7]	0.07[0.04–20.3]		0.3[0.1–2.4]	1.2[0.6–14.4]		1.1[0.3–4.8]	0.8[0.4–1.7]	0.6[0.2–6.1]		0.7[0.4–3.6]	0.9[0.07–9.9]	0.3[0.1–2.6]	0.7[0.5–13.8]		4.5[0.5–27.1]
Bronchial cell counts‡																					
**CD3^+^ cells**	118.5[45.0–171]	46.8[.9–92.0]		113.5[67.6–168.6]	25.0[15.5–37.8]	134.8[95.8–260.8]	57.3[36.6–86.3]		169.5[75.1–237.1]	28.0[15.3–43.5]		35.3[26.5–70.9]	26.8[12.0–54.8]	25.0[17.8–39.5]		16.5[12.3–60.0]	38.0[27.5–71.3]	69.5[33.4–90.9]	22.8[13.3–40.5]		18.8[4.5–43.5]
**CD4^+^ cells**	37.0[14.0–58.5]	27.8[17.1–66.1]		41.0[27.3–71.6]	10.3[6.4–17.1]	41.8[18.9–177.8]	36.3[7.4–54.6]		71.0[43.1–95.8]	11.0[7.3–20.3]		17.0[10.6–40.3]	11.0[8.0–33.5]	10.8[6.4–20.1]		14.0[6.5–25.5]	18.0[7.3–52.3]	29.5[25.5–43.5]	10.8[7.4–20.1]		15.0[9.5–33.5]
**CD8^+^ cells**	15.5[9.0–52.0]	13.8[8.9–32.6]		18.0[9.5–38.6]	8.0[4.9–10.4]	24.0[12.4–75.8]	12.5[7.3–18.3]		23.3[8.0–40.9]	5.5[3.0–8.5]		9.5[5.9–25.4]	11.0[4.3–31.0]	8.5[6.1–11.5]		9.3[2.4–14.1]	7.0[3.0–12.8]	15.5[10.3–25.0]	6.3[3.8–8.4]		3.5[1.9–6.1]
**Mast cells**	20.5[16.5–33.5]	11.8[8.6–15.6]		28.5[21.6–33.9]	8.0[5.9–10.25]	27.3[23.1–35.0]	11.3[7.1–12.5]		23.8[16.6–39.4]	5.0[2.0–8.0]		11.0[6.5–13.9]	13.3[7.1–15.9]	8.0[6.3–9.6]		5.3[2.3–10.1]	6.0[2.8–10.0]	12.8[11.0–18.8]	5.8[1.3–10.5]		1.3[0.0–3.0]
Sputum cell counts§																					
**Neutrophils**	43.7[20.2–89.4]	35.0[15.0–70.8]		79.5[31.2–146.6]	62.2[31.2–177.2]	158.0[89.2–215.1]	197.1[23.4–273.7]		118.8[68.5–340.6]	80.7[50.0–137.8]		50.2[19.4–142.8]	64.9[12.1–109.7]	62.2[22.4–177.2]		30.3[12.1–81.2]	77.9[32.9–234.2]	60.7[14.5–163.9]	86.5[52.3–144.9]		33.3[8.2–74.6]
**Eosinophils**	0.7[0.0–3.3]	0.4[0.0–1.2]		1.2[0.4–4.4]	0.9[0.3–3.0]	2.0[0.8–4.2]	0.9[0.3–7.1]		2.7[0.9–6.3]	0.9[0.08–2.4]		0.7[0.08–1.5]	0.6[0.2–2.6]	0.8[0.2–3.0]		0.5[0.1–2.9]	0.9[0.2–5.8]	0.5[0.0–1.1]	0.9[0.0–2.3]		0.3[0.1–1.0]
**Lymphocytes**	1.6[0.4–2.3]	0.7[0.2–1.9]		1.9[0.6–3.9]	2.2[0.6–4.3]	8.3[2.5–9.2]	6.6[1.4–11.0]		4.4[1.9–10.9]	3.0[0.6–6.5]		0.9[0.4–2.1]	1.2[0.3–3.1]	2.4[0.5–4.3]		0.7[0.1–1.7]	4.7[0.6–8.8]	2.0[0.8–4.3]	3.0[0.6–6.9]		1.2[0.2–2.5]
**Macrophages**	21.8[8.6–46.7]	10.9[8.3–21.8]		26.5[19.2–48.4]	31.4[13.4–52.2]	47.3[21.9–65.2]	56.7[8.1–59.0]		48.5[17.8–84.1]	24.4[13.5–48.3]		21.8[10.2–42.4]	17.6[4.4–35.9]	26.4[11.2–40.0]		7.3[2.7–25.1]	23.9[12.0–57.5]	18.3[9.9–42.7]	25.9[14.5–43.7]		12.9[5.4–19.3]

* Patients were selected from the GLUCOLD cohort [8]; only persistent smokers and ex-smokers were included. Values before and after treatment are presented of short-term ICS treatment (baseline and after 6 months), and long-term ICS treatment (6 months to 30 months).

Data are expressed as medians [Interquartile Ranges]; n = number; FEV_1_, post, L = FEV_1_ after salbutamol expressed in liters; PC_20_ = provocative concentration of metacholine causing a fall in FEV_1_ of >20%;

‡ Cell counts/10^−7^ per m^2^ of subepithelium;

§ Cell counts×10^4^ per mL.

### Short-term Effects

Multiple regression analyses showed that ICS treatment increased PC_20_ significantly in ex-smokers (Table 2). ICS treatment significantly reduced bronchial mast cells in both smokers and ex-smokers compared to placebo. Additionally, bronchial CD3^+^, CD4^+^, and CD8^+^ cells were significantly reduced by ICS treatment in smokers only (Table 2).There were no significant interactions between ICS treatment and smoking.

**Table 2:** Multiple regression analyses: effects of ICS treatment in smokers and ex-smokers, and the interaction between ICS treatment and smoking (smoking×ICS) on changes in lung function, hyperresponsiveness and inflammatory cells in biopsies and sputum.*

	0–6 months ICS treatment						6–30 months ICS treatment					
	Ex-smokers		Smokers		Smoking×ICS		Ex-smokers		Smokers		Smoking×ICS	
	*B*	*p*	*B*	*p*	*B*	*p*	*B*	*p*	*B*	*p*	*B*	*p*
**FEV_1_, post, L**	0.09	0.27	−0.07	0.27	−0.17	0.12	0.09	0.26	**0.14**	**0.04**	0.05	0.62
**PC_20_, mg/mL**	**2.65**	**0.02**	0.49	0.59	−2.16	0.13	**2.40**	**0.02**	0.41	0.68	−1.99	0.16
*Bronchial cell counts* ‡												
**CD3^+^ cells**	−0.66	0.13	**−0.92**	**0.01**	**−**0.27	0.63	**−**0.71	0.11	0.36	0.39	1.07	0.08
**CD4^+^ cells**	**−**0.86	0.07	**−1.37**	**0.00**	**−**0.51	0.40	**−**0.03	0.94	0.34	0.44	0.37	0.55
**CD8^+^ cells**	**−**0.55	0.21	**−0.70**	**0.05**	**−**0.15	0.79	**−1.08**	**0.02**	**−**0.03	0.96	1.06	0.11
**Mast cells**	**−0.83**	**0.00**	**−0.50**	**0.04**	0.33	0.37	**−1.36**	**0.00**	**−0.67**	**0.04**	0.69	0.17
*Sputum cell counts* §												
**Neutrophils**	**−**0.44	0.45	**−**0.04	0.93	0.40	0.59	**−1.24**	**0.04**	0.76	0.21	0.61	0.45
**Eosinophils**	**−**1.55	0.12	0.25	0.74	1.80	0.15	0.39	0.67	**−**0.60	0.44	**−**0.98	0.41
**Lymphocytes**	**−**0.14	0.85	**−**0.08	0.89	0.06	0.95	**−**1.30	0.13	**−**1.33	0.08	**−**0.03	0.98
**Macrophages**	**−**0.39	0.53	0.17	0.73	0.55	0.48	1.05	0.95	**−**13.99	0.34	**−**15.03	0.49

* Patients were selected from the GLUCOLD cohort [8]; only persistent smokers and ex-smokers were included. All analyses are adjusted for age and sex. Data are expressed as B, p (regression coefficient, p-value); significant data (p<0.05) are presented in bold; FEV_1_, post, L = FEV_1_ after salbutamol expressed in liters; PC_20_ = provocative concentration of metacholine causing a fall in FEV_1_ of >20%;

‡ Cell counts/10^−7^ per m^2^ of subepithelium;

§ Cell counts×10^4^ per mL. The absolute changes in bronchial cell counts and sputum cell counts were normalized by Ln-transformation.

### Long-term Effects

**Figure 1:**
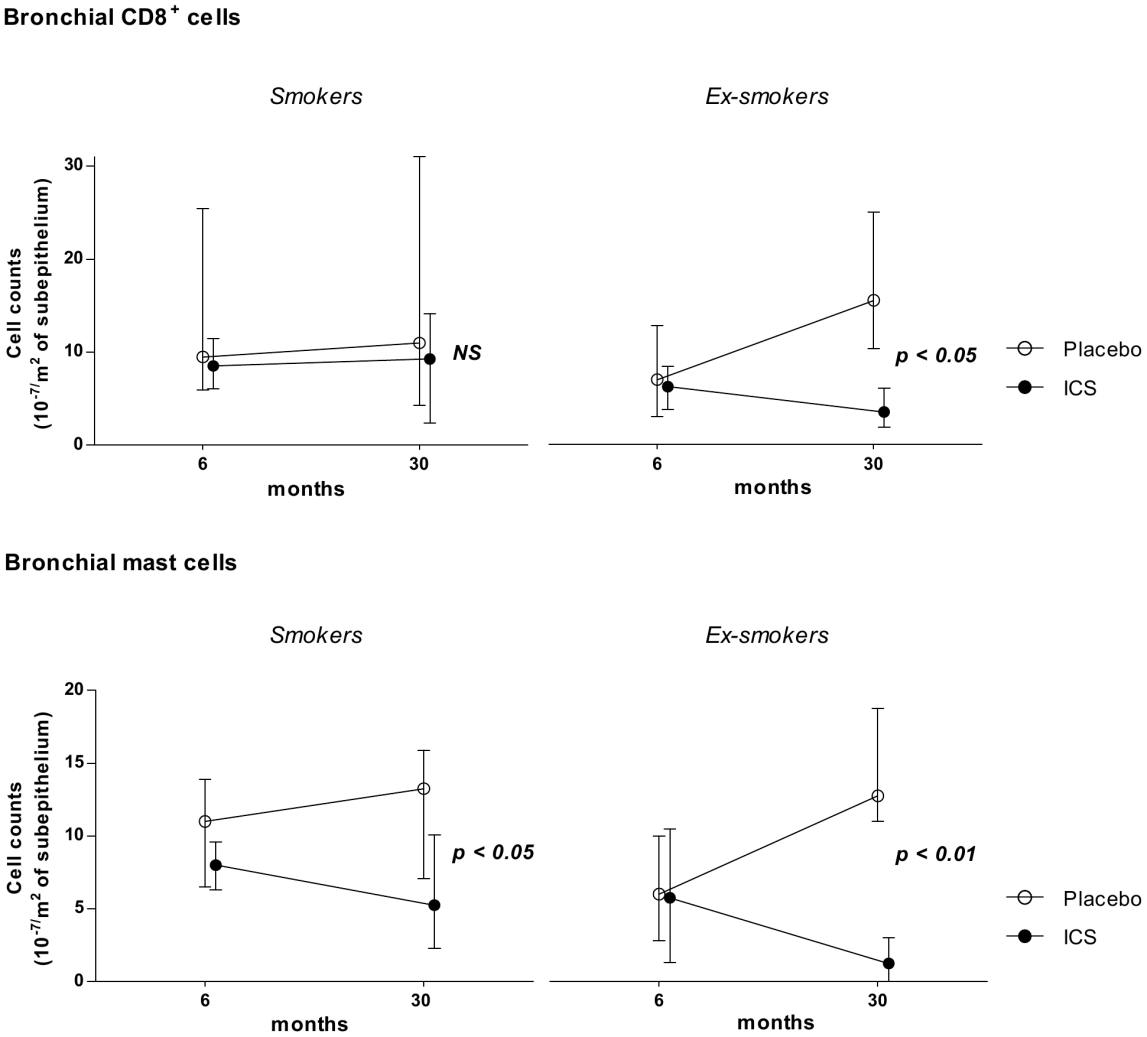
Effects of long-term ICS treatment on CD8^+^ cells and bronchial mast cells. Effects of ICS treatment from 6–30 months in persistent smokers and persistent ex-smokers on bronchial CD8^+^ cells (upper panel) and bronchial mast cells (lower panel). Data are presented as medians (Interquartile Ranges). NS = not significant.

Stratified analyses showed significant reductions in bronchial mast cells in both smokers and ex-smokers with ICS treatment compared to placebo (Table 2, Figure 1). In contrast to smokers, ex-smokers had significant reductions in bronchial CD8^+^ cells with ICS treatment as well as sputum neutrophil counts (Table 2, Figure 1). There existed no significant interactions between smoking and ICS treatment (Table 2).

## Discussion

This study investigated the confounding effect of concurrent smoking with respect to the short- and long-term effects of ICS treatment on clinical and inflammatory outcomes in persistent smokers and persistent ex-smokers with COPD participating in the GLUCOLD study. We did not find statistical interactions between ICS treatment and smoking, implying that the effect of ICS treatment on clinical and inflammatory parameters was comparable in smokers and ex-smokers. Multiple regression analyses showed that bronchial mast cell numbers significantly were reduced both in persistent smokers and persistent ex-smokers. This occurred with short- and long-term ICS treatment, showing the robustness of this observation. Furthermore, a significant reduction in number of bronchial CD8^+^ lymphocytes was observed after long-term ICS treatment in ex-smokers only. This is compatible with results in the main paper [8]. Additionally, stratified analysis showed that sputum neutrophils were reduced during long-term ICS treatment in ex-smokers only, a finding that fits published studies on steroid responsiveness [12]. Bronchial CD3^+^, CD4^+,^ and CD8^+^ cells were reduced in smokers only after short-term ICS treatment.

So far, studies investigating the effect of smoking on corticosteroid responsiveness have been performed in asthmatics predominantly. Smoking asthmatics were shown to be less sensitive to corticosteroids than non-smoking asthmatics [13]–[15]. There have been less studies published investigating the effect of smoking on corticosteroid responsiveness in COPD than in asthma. One short-term study (10-week treatment) found no anti-inflammatory effects of ICS treatment investigating inflammatory mediators like IL-8, IL-10, neutrophil elastase in sputum in smokers with COPD, whereas limited beneficial effects were present in ex-smokers [16]. In a study focusing on inflammation in airway wall biopsies, effects of ICS+salmeterol treatment were in the same direction in smokers and ex-smokers, although the reduction in cell counts (CD8^+^, CD4^+^, CD45^+^, mast cells, CD68^+^) were generally greater in former smokers [12]. Our data link, to our knowledge for the first time, the effects of short-term and long-term ICS treatment to the smoking status of patients with COPD. We show reductions particularly in mast cells in both persistent smokers and persistent ex-smokers, whereas other cell types were sometimes differentially affected in these two groups.

We believe that the fact that we analysed patient groups containing exclusively persistent smokers or persistent ex-smokers, in addition to the long-term intervention and the combined clinical and inflammatory outcomes represent the strengths of the current study. There are some limitations as well. We did not find significant interactions of ICS treatment response and smoking. This can be a real observation, yet it may also be due to low power, caused by the reduction in number of participants since we excluded switchers in smoking status. This resulted in small numbers in some subgroups, e.g. only 6 ex-smokers using ICS for six months, and thus this asks for caution of providing firm conclusions. For instance, there were differential effects with regard to short- and long-term treatment effects on lymphocytes, which may particularly result from analysing small numbers of patients. However, the direction of effects of other cell types was comparable with both short- and long-term follow-up providing robust findings. Additionally, we have to take into account that combining treatment groups in the 6–30 months period might have affected the results, for example by the effect of steroid withdrawal in the control group. However, when the individuals who stopped FP after 6 months were excluded, the analyses showed the same direction of effects and still reached significance despite lower study power (Table S1). We realize that the current study is the only one available with long-term biopsy data. There may have occurred some selection bias due to the strict definition of the patient groups. Notwithstanding this, the effects observed were, with respect to clinical (PC_20_, FEV_1_) and inflammatory parameters, in the same direction as the main results [8], and our stratified analyses confirmed the multiple regression findings. One may argue that there are baseline differences in cell counts in the various groups under study. Therefore, we analysed our data with adjustment of baseline cell counts as well and found that results of biopsy and sputum analyses were comparable. The (borderline) significant effects of long-term ICS treatment on neutrophils in the total group and ex-smokers became insignificant.

How can we explain that mast cell numbers were reduced by short- and long-term ICS treatment in smokers and ex-smokers in stratified analyses, whereas other cells were affected either in smokers or ex-smokers, or not affected at all, as for instance macrophages? One explanation may be that this reflects an epigenetic phenomenon [20] that may occur with DNA methylation and histone modification. Histone deacetylase-2 can be inactivated by oxidative and nitrative stress due to cigarette smoking [17] thereby inducing chromatin condensation and repression of gene transcription. In this way it can interfere with the anti-inflammatory actions of inhaled corticosteroids. Epigenetic regulation is known to be cell-specific and tissue-specific, selectively inactivating genes and signal transduction pathways [20]. This may thus explain differential effects of ICS in smokers and ex-smokers in specific cell types.

Gizycki et al. found that mast cells were reduced with 3-month ICS treatment in COPD patients who had high packyears smoking and smoked on average 27 cigarettes per day [21], corroborating our finding that mast cells are reduced by ICS treatment in persistent smokers with COPD. This suggests that epigenetic effects of smoking, if present, do not take place in mast cells with respect to the ultimate effects of ICS treatment. We found that bronchial CD8^+^ cells specifically were reduced in ex-smokers after long-term ICS treatment. An in vitro study by Kaur et al. on lymphocytes obtained by bronchoalveolar lavage in COPD patients showed that dexamethasone reduced IL-2 and INFgamma production after PHA/PMA stimulation [22]. Effects were comparable in smokers and ex-smokers, suggesting that activation of lymphocytes is steroid sensitive in COPD regardless of smoking. We found that CD8 cells were significantly reduced by long-term ICS treatment in ex-smokers and not in smokers with COPD. Although, the interaction between smoking and ICS treatment was not statistically significant, the estimated difference between the ex-smokers and current smokers with respect to the ICS-effect is striking. Whether these seemingly different observations are due to the relatively low number of patients under study in our investigation, to differential effects of steroids on lung influx and activation of lymphocytes, or to differences in steroid sensitivity when tested in all lymphocyte subtypes (as studied by Kaur et al) versus the CD8 subset in our study has yet to be determined. Our data together might reflect the idea that CD8^+^ cells, which are important key players in COPD, are sensitive to e.g. histone modification induced by smoke exposure, whereas mast cells are not.

We suggest that the clinical implication of our findings may be that, even in the presence of smoking, long-term ICS treatment may lead to anti-inflammatory effects in the lung. However, especially in COPD patients with frequent exacerbations, the balance between positive clinical and anti-inflammatory effects and side effects (e.g. pneumonia [7]) still has to be considered. We put forward the hypothesis that steroid unresponsiveness in COPD is not a general characteristic. Some anti-inflammatory ICS effects are comparable in smokers and ex-smokers with COPD, yet other effects are cell-specific, dependent on smoking status. Studies in larger datasets are needed to prospectively study the underlying mechanisms of similar or differential ICS effects in smokers and ex-smokers with COPD. This will open avenues for further targeted therapy in persistent smokers and ex-smokers with COPD.

## Supporting Information

Table S1 **Patients were selected from the GLUCOLD cohort** [**7**] **; only persistent smokers and ex-smokers were included.** All analyses are adjusted for age and sex. Data are expressed as B, p (regression coefficient, p-value); significant data (p<0.05) are presented in bold; FEV_1_, post, L = FEV_1_ after salbutamol expressed in liters; PC_20_ = provocative concentration of metacholine causing a fall in FEV_1_ of >20%; ‡ Cell counts/10^−7^ per m^2^ of subepithelium; § Cell counts×10^4^ per mL. The absolute changes in bronchial cell counts and sputum cell counts were normalized by Ln-transformation.
(DOC)
