# Supplementary material for: Steroid Resistance in COPD? Overlap and Differential Anti-Inflammatory Effects in Smokers and Ex-Smokers
Source: PLoS One. 2014 Feb 5;9(2):e87443. doi: 10.1371/journal.pone.0087443 (PMC3914834; doi:10.1371/journal.pone.0087443)
Supplement: Table S1 — Patients were selected from the GLUCOLD cohort [7] ; only persistent smokers and ex-smokers were included. All analyses are adjusted for age and sex. Data are expressed as B, p (regression coefficient, p-value); significant data (p<0.05) are presented in bold; FEV1, post, L = FEV1 after salbutamol expressed in liters; PC20 = provocative concentration of metacholine causing a fall in FEV1 of >20%; ‡ Cell counts/10−7 per m2 of subepithelium; § Cell counts×104 per mL. The absolute changes in bronchial cell counts and sputum cell counts were normalized by Ln-transformation. (DOC) [file pone.0087443.s001.doc]

**Steroid resistance in COPD?**

**Overlap and differential anti-inflammatory effects in smokers and ex-smokers**

S.J.M. Hoonhorst^1,2^, N.H.T. ten Hacken^1,2^, J.M. Vonk^2,3^, W. Timens^4,2^, P.S. Hiemstra^5^,

T.S. Lapperre^5^, P.J. Sterk^6^*,* D.S. Postma^1,2^

**Online supplement**

**Table 1.**

|  | **FP 6-month treatment included**  6-30 months ICS treatment | | | | | | **FP 6-month treatment excluded**  6-30 months ICS treatment | | | | | |
| --- | --- | --- | --- | --- | --- | --- | --- | --- | --- | --- | --- | --- |
|  | **Ex-smokers** | | **Smokers** | | **Smoking x ICS** | | **Ex-smokers** | | **Smokers** | | **Smoking x ICS** | |
|  | *B* | *p* | *B* | *p* | *B* | *p* | *B* | *p* | *B* | *p* | *B* | *p* |
| **FEV_1_, post, L** | 0.09 | 0.26 | **0.14** | **0.04** | 0.05 | 0.62 | 0.09 | 0.43 | **0.18** | **0.03** | 0.09 | 0.48 |
| **PC_20_, mg/mL** | **2.40** | **0.02** | 0.41 | 0.68 | -1.99 | 0.16 | -0.05 | 0.98 | 0.09 | 0.94 | 0.14 | 0.94 |
| *Bronchial cell counts ‡* |  |  |  |  |  |  |  |  |  |  |  |  |
| **CD3^+^ cells** | -0.71 | 0.11 | 0.36 | 0.39 | 1.07 | 0.08 | -0.57 | 0.35 | 0.89 | 0.11 | 1.45 | 0.08 |
| **CD4^+^ cells** | -0.03 | 0.94 | 0.34 | 0.44 | 0.37 | 0.55 | 0.15 | 0.78 | 1.02 | 0.04 | 0.87 | 0.23 |
| **CD8^+^ cells** | **-1.08** | **0.02** | -0.03 | 0.96 | 1.06 | 0.11 | **-1.33** | **0.02** | 1.20 | 0.06 | 1.70 | 0.03 |
| **Mast cells** | **-1.36** | **0.00** | **-0.67** | **0.04** | 0.69 | 0.17 | **-1.13** | **0.03** | **-0.87** | **0.04** | 0.26 | 0.69 |
|  |  |  |  |  |  |  |  |  |  |  |  |  |
| *Sputum cell counts §* |  |  |  |  |  |  |  |  |  |  |  |  |
| **Neutrophils** | **-1.24** | **0.04** | 0.76 | 0.21 | 0.61 | 0.45 | **-0.75** | **0.40** | -1.25 | 0.06 | -0.50 | 0.64 |
| **Eosinophils** | 0.39 | 0.67 | -0.60 | 0.44 | -0.98 | 0.41 | 1.51 | 0.25 | -1.38 | 0.15 | -2.89 | 0.08 |
| **Lymphocytes** | -1.30 | 0.13 | -1.33 | 0.08 | -0.03 | 0.98 | -0.90 | 0.48 | -2.00 | 0.04 | -1.10 | 0.49 |
| **Macrophages** | 1.05 | 0.95 | -13.99 | 0.34 | -15.03 | 0.49 | 6.12 | 0.79 | -24.16 | 0.15 | -30.28 | 0.28 |

Patients were selected from the GLUCOLD cohort[7]; only persistent smokers and ex-smokers were included.

All analyses are adjusted for age and sex. Data are expressed as B, p (regression coefficient, p-value); significant data (p<0.05) are presented in bold; FEV_1_, post, L = FEV_1_ after salbutamol expressed in liters; PC_20_ = provocative concentration of metacholine causing a fall in FEV_1_ of > 20%; ‡ Cell counts / 10^-7^ per m^2^ of subepithelium; § Cell counts x10^4^ per mL. The absolute changes in bronchial cell counts and sputum cell counts were normalized by Ln-transformation.
